# Growth and rupture of an intracranial aneurysm: the role of wall aneurysmal enhancement and CD68+

**DOI:** 10.3389/fsurg.2023.1228955

**Published:** 2023-09-06

**Authors:** Delia Cannizzaro, Ismail Zaed, Simone Olei, Bethania Fernandes, Simone Peschillo, Davide Milani, Andrea Cardia

**Affiliations:** ^1^ Department of Neurosurgery, IRCCS Humanitas Research Hospital, Milan, Italy; ^2^ Department of Biomedical Sciences, Humanitas University, Milan, Italy; ^3^ Department of Neurosurgery, Neurocenter of South Switzerland, Ente Ospedaliero Cantonale, Lugano, Switzerland; ^4^ Department of Pathology, IRCCS Humanitas Research Hospital, Milan, Italy; ^5^ Unicamillus-Saint Camillus International University of Health Sciences, Rome, Italy

**Keywords:** aneurysms, inflammation, vessel wall, wall enhancement, vascular malformation, technique, hemorrhage

## Abstract

**Introduction:**

Intracranial aneurysms occur in 3%–5% of the general population. While the precise biological mechanisms underlying the formation, growth, and sudden rupture of intracranial aneurysms remain partially unknown, recent research has shed light on the potential role of inflammation in aneurysm development and rupture. In addition, there are ongoing investigations exploring the feasibility of employing new drug therapies for controlling the risk factors associated with aneurysms. CD68, a glycosylated glycoprotein and the human homolog of macrosialin, is prominently expressed in monocyte/macrophages within inflamed tissues and has shown potential application in oncology. An observational study was conducted with the aim of comparing the histological characteristics of aneurysm walls with preoperative MRI scans, specifically focusing on CD68 activity.

**Method:**

An observational pilot study was conducted to investigate the histological characteristics of the aneurysm wall that could be potentially associated with aneurysm growth and rupture. A total of 22 patients diagnosed with ruptured and unruptured intracranial aneurysms who had undergone conventional clipping between January 2017 and December 2022 were included in the study.

**Results:**

A histopathological analysis of the aneurysm wall was performed in all patients, particularly focusing on the presence of CD68. A preoperative MRI with gadolinium was conducted in 10 patients with unruptured aneurysms and six patients with ruptured aneurysms. An emergency clipping was performed in the remaining six patients. The results showed that CD68 positivity and wall enhancement were significantly associated with intracranial aneurysm wall degeneration, growth, and rupture.

**Conclusion:**

The histological and radiological inflammatory findings observed in the wall of cerebral aneurysms, as well as the CD68 positivity, are significantly associated with the risk of intracranial aneurysm growth and rupture. This study highlights the crucial importance of considering clinical and medical data when making treatment decisions for intracranial aneurysms. Furthermore, it emphasizes the relevance of evaluating wall enhancement in MRI scans as part of the diagnostic and prognostic process.

## 1. Introduction

Unruptured intracranial aneurysm (UIA) in the general population has an incidence rate estimated to be between 3% and 5% (1). The prevalence rate of UIAs is higher than that of ruptured aneurysms (2), and only 27% of patients diagnosed with a UIA will suffer from a subarachnoid hemorrhage (SAH) (3). The advancements in non-invasive imaging techniques lead to increasing the detection of UIAs in clinical practice. Identifying aneurysms with high risk of rupture is crucial for determining appropriate management strategies. Hemorrhagic risk stratification has traditionally relied on analyzing known risk factors and aneurysm sac morphology and size (4–6). However, recent research has shown a growing interest in the role of inflammation in the natural history of intracranial aneurysms (IAs). Radiological imaging reports have provided new evidence supporting the involvement of inflammatory mechanisms in aneurysm growth and rupture (7). Consequently, there has been a surge in interest in studying the walls of cerebral aneurysms. Specifically, endothelial dysfunction is recognized as the primary factor contributing to aneurysm sac growth, with certain factors such as cigarette smoking and hypertension associated with this dysfunction (8). After the initial endothelial injury, the inflammation cascade is activated, leading to characteristic changes in the layers of the aneurysm sac (9). CD68, a glycosylated glycoprotein, is abundantly expressed in macrophages and other phagocytic cells (10). It has been observed in histological examinations of intracranial aneurysms, and it serves as an immunohistochemical marker for inflamed tissue, tumors, and other applications (11). Several studies have investigated the presence of CD68 in the histological examination of intracranial aneurysms (12), building upon these recent discoveries of risk factors for the development and rupture of intracranial aneurysms and histological changes in the aneurysm wall in patients undergoing surgical clipping.

In addition, the study correlated histological findings with radiological imaging supported by a thorough literature review. The primary objective of this study and review was to examine the role of inflammation in managing intracranial aneurysms, emphasizing the importance of analyzing the risk factors and radiological aspects for risk stratification.

## 2. Materials and methods

### 2.1. Methods design

This study was designed as an observational study in accordance with the principles outlined in the Declaration of Helsinki.

### 2.2. Patient eligibility

Adult patients who had undergone intracranial clipping for either ruptured or unruptured aneurysms were included in this study. The inclusion period spanned 60 months from January 2017 to December 2022. Before participation, an informed consent for the surgical procedure and data processing was obtained from each patient according to the guidelines set by the Institutional Review Board.

### 2.3. Inclusion and exclusion criteria

The following criteria were required for inclusion in the study: (1) age ≥18 years, (2) had undergone surgical procedures for ruptured or unruptured intracranial aneurysms, and (3) with radiological diagnosis of intracranial aneurysms obtained through digital subtraction angiography (DSA), magnetic resonance angiogram (MRA), or three-dimensional computed tomography angiogram (3D-CTA). The indication for surgical treatment was determined following a multidisciplinary discussion involving a neurosurgeon and neurointerventional radiologist, who analyzed each case. The following criteria led to exclusion from the study: (1) age <18 years; (2) incomplete clinical data; (3) with fusiform, traumatic, or mycotic aneurysms; (4) with a previous diagnosis of autoimmune or rheumatological diseases; (5) with ongoing systemic infections; and (6) pregnant.

### 2.4. Data collection

The examiner conducted interviews with the patients and collected relevant data from their medical records. The study coordinator reviewed the collected data and made decisions regarding the inclusion of each patient in the study. Various clinical and radiological factors were considered in this research, including the age and sex of the patient, whether the aneurysm had ruptured or was unruptured, any previous diagnosis or history of rupture of an IA, presence of multiple IAs, IA location, positive familial history of IA or SAH, hypertension, dyslipidemia, diabetes, smoking status, alcohol abuse, a radiological sign of single or multiple blebs, and a surgical evidence of sac thrombosis or calcification. For patients with UIAs and those with good-grade aneurysmal subarachnoid hemorrhage, a 3T magnetic resonance scanner using T1-weighted imaging with gadolinium was utilized to identify the presence of aneurysm wall enhancement.

### 2.5. Histological analysis

A descriptive histological evaluation was conducted by a single pathologist (BF) at the Department of Pathology of Humanitas University. The histological analysis was performed on a full-thickness fragment of the aneurysm dome after conventional clipping. The aneurysm domes were fixed in paraffin, sectioned at 5 µm, and conserved at 4°C. The sections were stained with hematoxylin and eosin for a general histological examination. In addition, immunochemistry was performed using antibodies specific to CD68 and glial fibrillary acidic protein (GFAP) to assess their expression in the tissue sections.

### 2.6. Study organization

The steering board of the study consisted of the principal investigator, coordinating investigator, and the local examiner for each participating department. The principal investigator took charge of designing, conducting, and reviewing the original study. The coordinating investigator was responsible for the administrative management of the study. The local examiner played a crucial role in scheduling data collection and ensuring the integrity of the data collection process.

### 2.7. Statistical analysis

The continuous distribution of the data was assessed by a visual inspection of histograms, which allowed for an examination of the frequency distribution of the variables. The baseline characteristics of the study participants were presented as counts and percentages for categorical variables, while the means and medians were used for continuous variables.

## 3. Results

### 3.1. Patient population

The patient population for this observational study consisted of 22 participants recruited between January 2017 and December 2022. There were nine male and 13 female patients. The surgeries for intracranial aneurysm clipping were performed at the Department of Neurosurgery at IRCCS Humanitas Research Hospital in Rozzano, Milan, Italy, and Neurocenter of South Switzerland, Ente Ospedaliero Cantonale, Lugano, Switzerland. The detailed patient and aneurysm characteristics are summarized in Table 1. The mean age of the population was 57.13 years, ranging 31–77 years old (SD 12.21). The majority of the aneurysms (14 cases, 63.6%) were located in the middle cerebral artery (MCA), followed by four cases (18.2%) located in the anterior communicating artery (ACoA), three cases (13.6%) located in the anterior cerebral artery (ACA), and one case (4.5%) located in the internal carotid artery (ICA). Among the patients, 12 patients presented with a ruptured aneurysm upon admission. The average diameter of the aneurysms, as measured on angiographic studies, was 8.68 mm, ranging from 5 to 12 mm (SD 1.62 mm). Calcification of the aneurysm wall was observed in 10 cases (45.5%), while thrombotic features were present in 12 patients (54.5%). In terms of comorbidities, 16 patients (72.7%) had hypertension, eight had dyslipidemia, and seven had type II diabetes mellitus (DM). In addition, 12 patients (54.5%) were current smokers (with an average use of 20 cigarettes per day), and 12 (54.5%) were alcohol abusers. Among the patients, 12 (54.5%) presented signs of SAH. Three patients (13.6%) had genetic syndromes associated with the development of an aneurysm. It is important to note that no statistical correlation (*p* = 0.67) was found between SAH, familial history of aneurysms, and activation of CD68+.

**Table 1:** Demographic and clinical data of patients included in the observational study.

*N*	Sex	Age	Location	SAH	Hypertension	Dyslipidemia	Diabetes	Smoking	Alcohol	Genetic syndromes	Size, mm	Growing 1 year	Calcifications	Thrombosis	CD68	Wall enhancement
1	M	55	MCA	Y	Y	Y	N	N	Y	N	7	—	N	Y	Y	—
2	F	52	MCA	Y	Y	Y	N	Y	Y	N	8	—	N	Y	Y	Y
3	F	64	ACoA	Y	Y	Y	Y	N	Y	N	5	—	Y	N	Y	Y
4	F	72	ACoA	Y	Y	N	Y	Y	Y	N	8	—	N	Y	Y	Y
5	F	44	ACA	Y	Y	N	Y	N	N	N	7		Y	Y	Y	Y
6	M	45	MCA	Y	Y	N	N	Y	N	N	9	—	Y	N	N	Y
7	M	38	MCA	Y	Y	N	N	Y	Y	N	11		N	Y	Y	Y
8	F	55	MCA	Y	Y	N	N	Y	N	N	11	—	N	N	Y	—
9	M	66	ACoA	Y	Y	Y	Y	N	Y	N	7	—	Y	N	N	Y
10	M	60	MCA	Y	Y	N	N	Y	N	Y	7	—	N	N	N	—
11	M	59	MCA	Y	N	N	N	N	N	N	10	—	Y	Y	N	—
12	F	67	ACA	Y	Y	Y	Y	Y	Y	N	9	—	Y	Y	Y	—
13	F	40	MCA	N	N	N	N	Y	N	N	8	N	N	N	N	—
14	F	50	MCA	N	N	N	N	Y	N	N	9	Y	N	Y	Y	Y
15	F	58	ICA	N	Y	N	N	N	N	N	8	N	Y	N	N	N
16	F	31	ACoA	N	N	N	N	Y	Y	N	12	Y	N	Y	Y	Y
17	F	66	MCA	N	Y	N	N	N	N	Y	9	Y	N	N	N	N
18	M	77	MCA	N	N	N	N	Y	Y	Y	8	Y	Y	Y	Y	Y
19	M	65	MCA	N	Y	Y	N	Y	Y	N	10	—	Y	N	N	N
20	F	73	ACA	N	Y	Y	Y	N	N	N	9	Y	Y	Y	Y	Y
21	M	68	MCA	N	N	N	N	N	Y	N	9	N	N	N	N	Y
22	F	52	MCA	N	Y	Y	Y	N	Y	N	10	Y	N	Y	Y	Y

### 3.2. Histological characteristics

**Figure 1:**
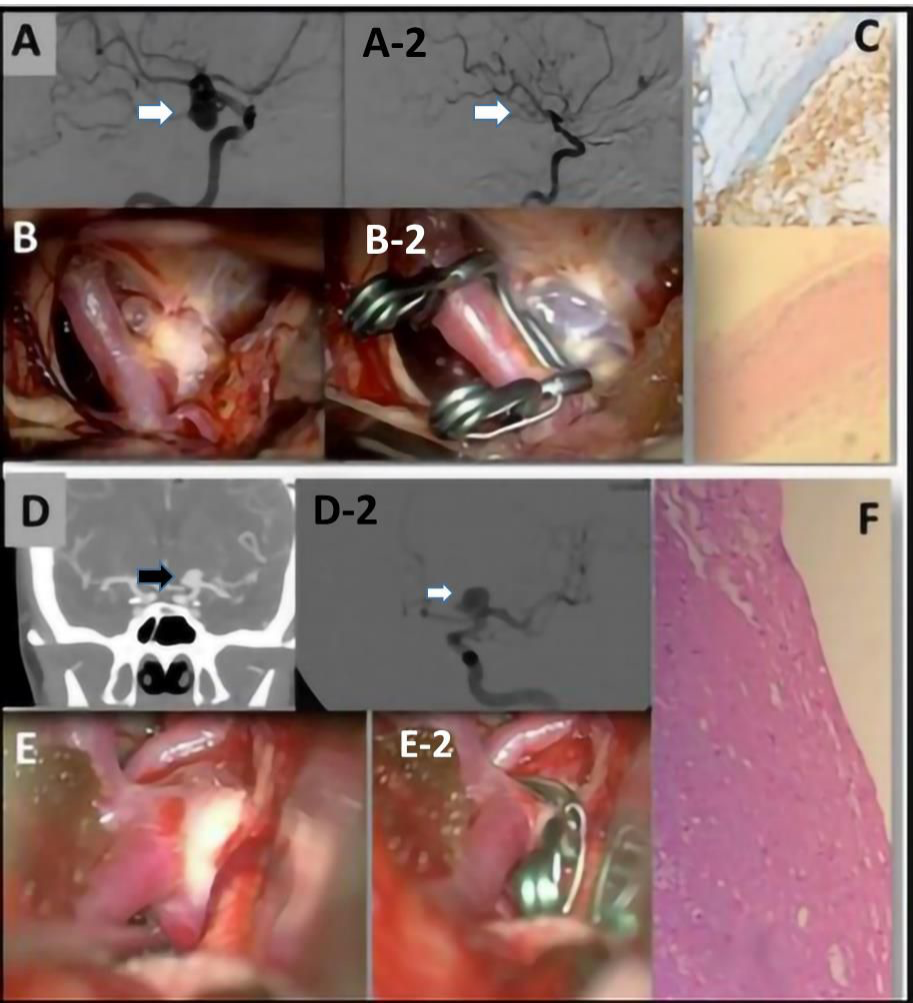
(**A**) A preoperative evaluation of an ICA aneurysm with a DSA. (**A**-2) A postoperative evaluation after clipping of the previous ICA aneurysm. (**B**) Image obtained with a surgical microscope of the intracranial aneurysm (IA). (**B**-2) Postoperative image obtained with a surgical microscope of the IA after clipping. (**C**) Representative examples of H&E staining performed on IA domes directly fixed in formalin. The aneurysm wall is characterized by fibrosis and foam cells. CD68+ foam cells. (**D**) A preoperative CTA image of a left ICA aneurysm and (**D**-2) a DSA evaluation of the same aneurysm. (**E**) Intraoperative view of the IA obtained before clipping and (**E**-2) obtained after the clipping. (**F**) Increase of the ECM associated with widespread rarefaction of the muscular component. In the adventitia, there is also lymphocytic inflammatory infiltrate and granulocytes.

**Figure 2:**
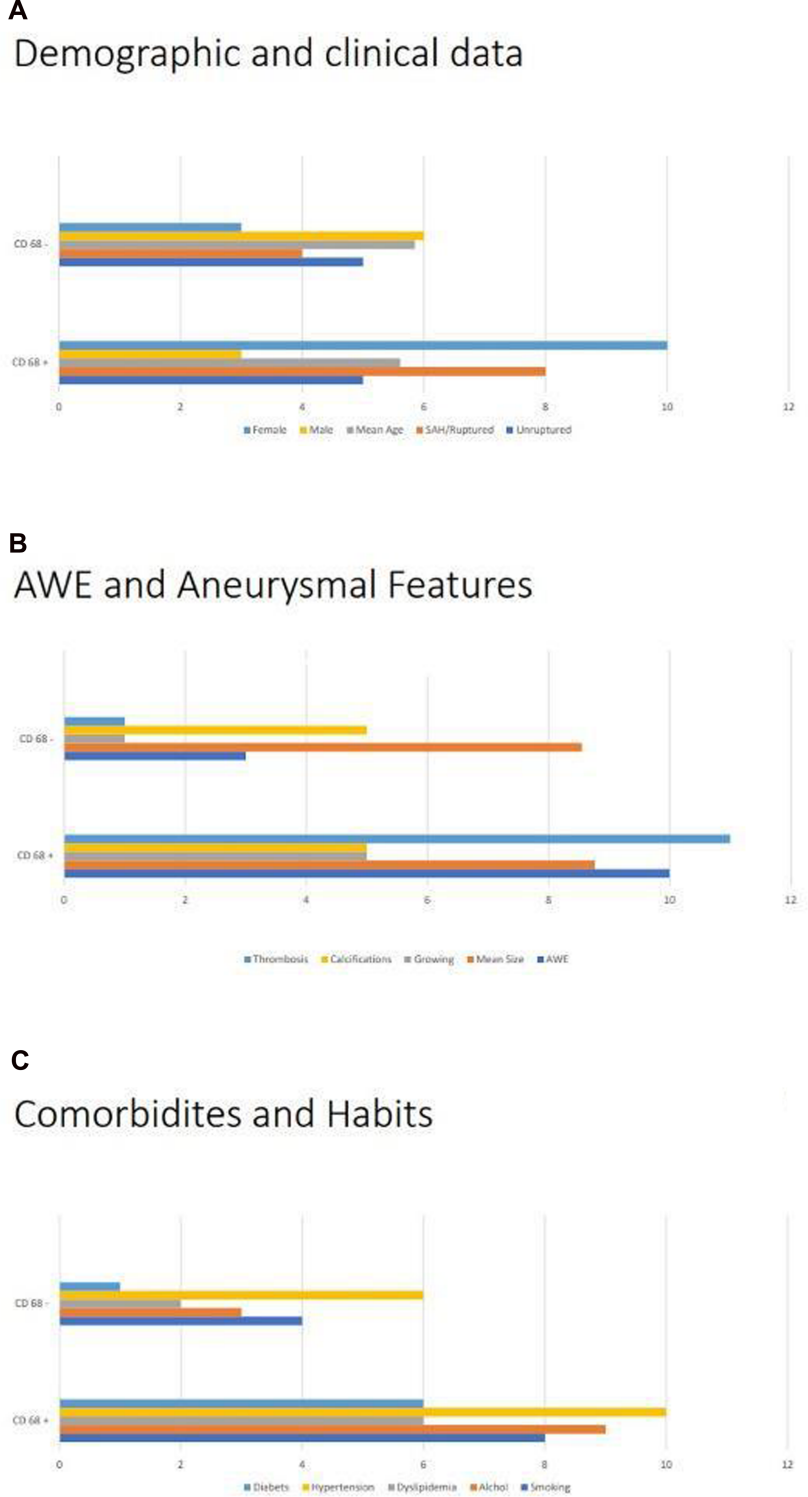
Analysis of CD68+: (**A**) comparison of CD68+ with demographic, namely, gender and mean ages, and clinical data, in particular the ruptures/unruptured status of the aneurysm. (**B**) Comparison of CD68+ with different radiological factors, such as growth state, mean size, and aneurysm wall enhancement, and histopathological features, in particular signs of thrombosis and calcifications. (**C**) Comparison of CD68+ comorbidities and habits, such as smoking status, consumption of alcohol, dyslipidemia, hypertension, and diabetes.

**Figure 3:**
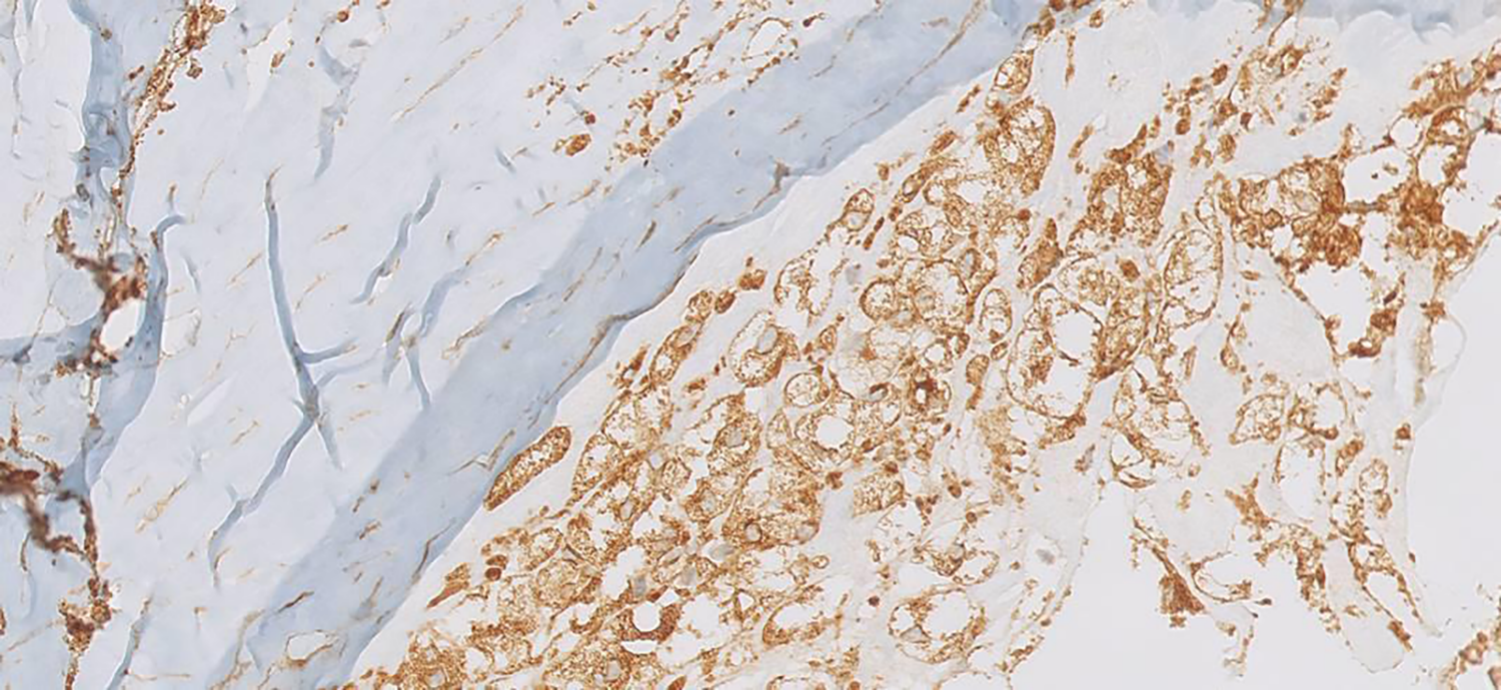
An example of a positive assay for CD68.

A total of 13 patients were identified showing positive effects for CD68 due to the composition of the aneurysm wall. In these patients, the aneurysm wall exhibited characteristics such as the presence of foaming elements of a macrophagic nature (foam cells), an increase in the extracellular matrix (ECM) in the tunica media; lymphocytic inflammatory infiltrate; and granulocytic neutrophils in the adventitia (Figure 1). On the other hand, in nine patients, relevant aspects of fibrosis and fibroblastic activation were observed, along with discrete aspects of elastic fiber fragmentation in the aneurysm wall. Figure 2 presents graphs depicting the main prevalence of CD68 parameters. These patients showed no effects for CD68 (Figure 3). Positive effects for CD68 were associated with higher prevalence of female patients, ruptured aneurysms, and subarachnoid hemorrhage. Other associated factors included thrombosis in the aneurysm wall, aneurysm wall enhancement, growth of the aneurysmatic sac at 1 year of radiological follow-up, diabetes, dyslipidemia, hypertension, and smoking and alcohol habit.

### 3.3. Radiological assessment and literature review

**Figure 4:**
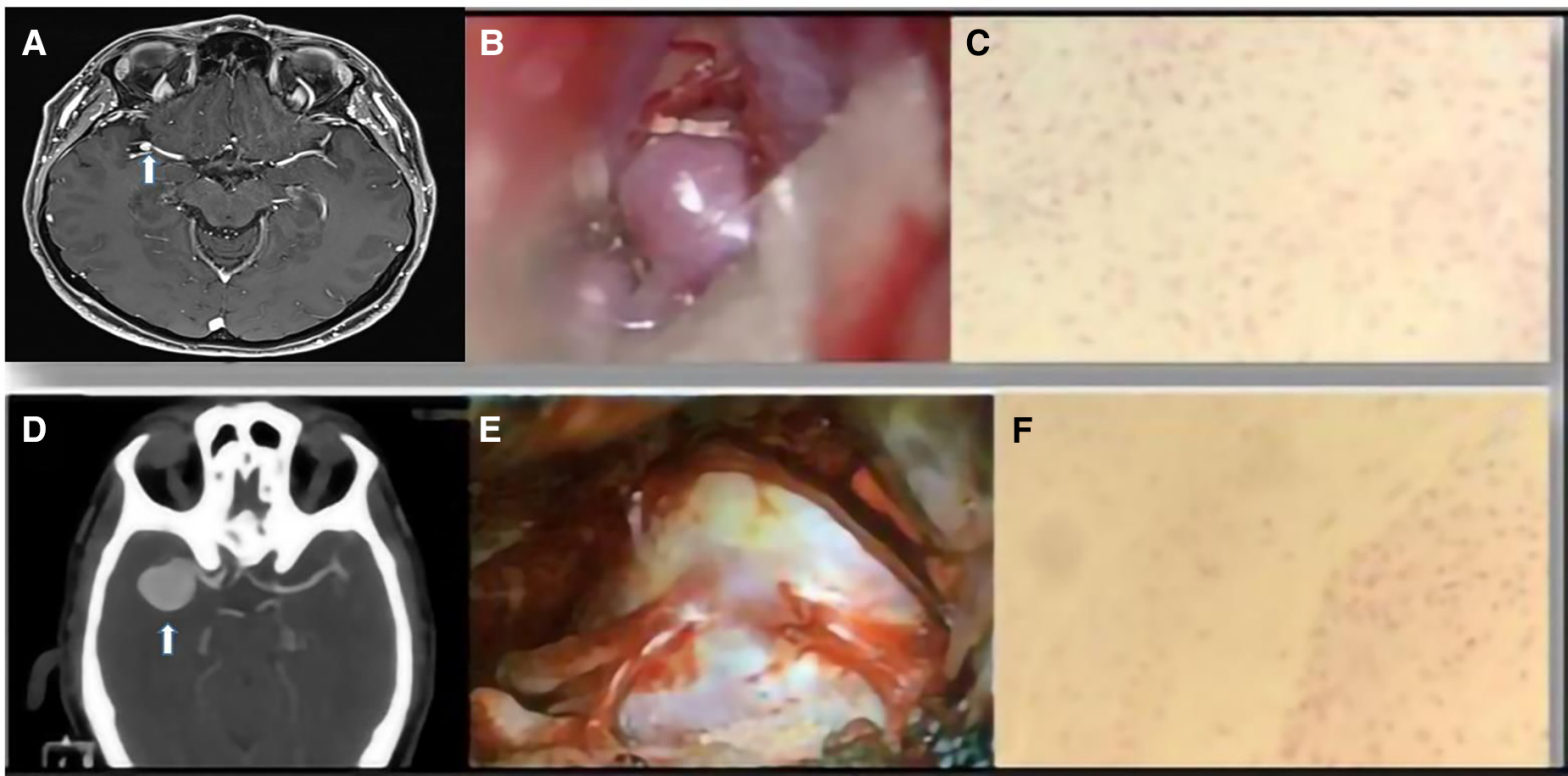
(**A**) An MRI image of an MCA aneurysm. (**B**) Intraoperative view. (**C**) Representative examples of fibrosis and fibroblastic activation in the aneurysm wall (H&E stain). (**D**) CTA image of a right MCA aneurysm. (**E**) Intraoperative view. (**F**) Focal areas of fibrosclerosis in the vascular wall (H&E stain).

Aneurysm wall enhancement was analyzed in 16 patients with UIAs. Among these patients, 13 showed vessel wall enhancement on MRI images (Figure 4). In 10 of these patients, there was a correlation between wall enhancement on MRI and similar histological findings, including the presence of foamy cells, lymphocytic inflammatory infiltrate, granulocytic neutrophils in the adventitia, and positive assays for CD68. Such correlation has not been supported by statistical significance (Figures 1, 3, 4).

## 4. Discussion

Despite the increasing interest in understanding the natural history of IAs, there are still many unknown aspects. The significant role of inflammation in the development and growth of IAs has been well-established and has primarily been investigated through animal studies (13–15). Inflammation is a natural response of tissue to chemical, biological, or mechanical damage, characterized by the accumulation of inflammatory cells and immune response. The potential involvement of inflammation in the development of intracranial aneurysms was first suggested by Virchow in 1847 (16). In the 1930s, Maas et al. (17) published further studies describing the presence of infiltrating round cells, possibly lymphocytes, predominantly localized at the neck of the aneurysm. Subsequent studies have also demonstrated the presence of inflammatory infiltrates within the wall of cerebral aneurysms, showing a strong association between these cells and aneurysm rupture (18, 19). Supporting these findings, mycotic aneurysms exhibit a local inflammatory reaction in response to the pathogen, highlighting the significant role of local inflammation in the process of tissue destruction (20–22). Indeed, the histological characterization of the cerebral aneurysm wall includes not only inflammatory cells but also other components such as fibroblasts, granulocytic neutrophil infiltrates, and frothy macrophages (23). Fibrosis is commonly regarded as the final phase of the chronic inflammatory process. Inflammatory cells have been identified below the endothelium and deep within the wall of IA, including polymorphonuclear leukocytes, plasma cells, and small round cells, as observed by Crompton (24). CD68 and GFAP (25) are highly correlated with circulating macrophages, which are typically involved in the inflammatory mechanisms of IA. In recent times, it has been highlighted by various authors (19–23, 26, 27) that the percentage of CD68-positive cells in recently ruptured aneurysm domes (within 2 days) is significantly higher compared with the percentage in unruptured aneurysm domes. The presence of foaming elements of macrophagic nature (foam cells) indicates a high concentration of lipid material, similar to that seen in vessels with atherosclerotic plaques, along with an inflammatory process in the aneurysm wall.

In our series, we observed that positive effects for CD68, in line with previous literature reports (27–29), were associated with a high percentage of SAH. These patients exhibited an increase in ECM along with rarefaction of the muscular component through the aneurysm wall. In addition, there was evidence of lymphocytic inflammatory infiltrate and granulocytic neutrophilic in the adventitia. It is noteworthy that these patients were receiving pharmacological treatment for hypertension, which could potentially explain both the atherosclerotic degeneration observed and the hemodynamic stress placed on the aneurysm wall.

Fibrosis is associated with a chronic process and the presence of multiple aneurysms. In our series, we also observed higher prevalence of female sex among patients with fibrosis. Dyslipidemia and type II DM can contribute to a chronic inflammatory process, thrombosis, and developing calcification in the wall of the aneurysmal sac. The presence of calcification in the neck of the aneurysm can pose challenges during surgical clipping due to mechanical limitations in achieving a proper clip closure (30). Bleb formation in the arterial walls, often observed in smoking patients, is characterized by the presence of fibrous connective tissue and thrombosis. These blebs are considered to be at higher risk of rupture, including intraoperatively (31, 32). Cigarette smoking (33) is a recognized common risk factor for IAs due to the role of inflammation associated with nicotine. Patients with familial genetic syndromes, such as polycystic kidney disease, and those with a positive family history of SAH (a first-degree relative with a history of SAHs) exhibit higher levels of fibrosclerosis, disruption elastic fibers, and chronic inflammatory infiltration, indicating persistent injury at the level of the aneurysm wall (34, 35). To evaluate the risk of aneurysm rupture, clinicians have compared patients and aneurysm characteristics in different outcome groups within large cohorts. They have identified the risk factors and developed scoring systems that integrate multiple variables, such as the International Study of Unruptured Intracranial Aneurysms (ISUIA) (36), Unruptured Cerebral Aneurysm Study (UCAS) (37), PHASES score (38), and the Unruptured Intracranial Aneurysm Treatment Score (UIATS) (39). These scores provide estimates of rupture probability based on various factors. Histological studies play a crucial role in enhancing our understanding of the mechanisms and progression of IAs. Achieving consensus on treatment standards is essential to make rapid progress in the field. Our study contributes to this goal by utilizing histological analysis to identify patient groups with similar clinical and histological features. The development of IAs is a complex process. Various factors are recognized as risks for aneurysm rupture or growth. The rupture of an aneurysm is possibly related to the hemodynamic stress induced by arterial hypertension, which can be present in any patients. Conversely, the presence of thrombotic phenomena in the neck and the sac of aneurysms may have a protective effect against rupture but can influence the growth of the aneurysmal sac, as observed in our study. Smoking and alcohol habit (40), arterial hypertension, and dyslipidemia are among the factors that influence the natural history of IA.

Larsen et al. (41) suggested a correlation between wall enhancement on imaging and inflammatory histological changes in the aneurysm wall. Similarly, Shimonaga et al. (42) confirmed that wall enhancement is related to histological aspects of inflammation and prominent macrophage infiltration. On the other hand, Hu et al. (43) found a correlation between wall enhancement and symptomatic aneurysms. Lv et al. (44) and Liu et al. (45) emphasized the correlation between the size and location of IAs and radiological findings. Recent meta-analyses (46, 47) have further supported the association between aneurysm rupture/instability and vessel wall enhancement observed on magnetic resonance imaging. Our descriptive literature review of radiological cases series strengthens the existing evidence and underscores the importance of evaluating the radiological characteristics of a UIA in defining the risk profile. Our study aimed to identify prognostic factors for aneurysm rupture and growth by integrating clinical data with histological and radiological analysis. These associations we found between available clinical, pathological, and radiological data, along with patients’ habits and detailed histological features, highlight the significance of carefully considering clinical and radiological data in the decision-making process for these vascular diseases.

### 4.1. Study limitations

Despite the authors’ best efforts, the study has several limitations. The most significant limitation is the relatively small number of patients included in this observational analysis. Due to the limited sample size, conducting an extensive statistical analysis was not feasible, so we performed a descriptive analysis of our preliminary results to establish the basis for our hypothesis. Another potential limitation is the bias related to the technique used for aneurysm wall removal. However, this bias was minimized by ensuring that experienced neurosurgeons with expertise in vascular disease performed the procedures. In addition, to minimize bias related to histological diagnosis, all histological analyses were conducted by a single pathologist. Future studies will help strengthen the findings and provide more conclusive evidence.

## 5. Conclusion

The natural history of both ruptured and unruptured cerebral aneurysms remains a subject of ongoing discussion within the scientific community. The role of inflammation in the development and rupture of aneurysms has been well-documented. In this observational study, we aim to investigate the potential correlation between clinical and medical history and the histological characteristics of cerebral aneurysm walls. Specifically, we emphasize the influential role of inflammation, as indicated by CD68 analysis, which is influenced by factors such as smoking habits, severe dyslipidemia, and subsequent macrophage infiltration. These factors may predispose aneurysms to rupture. Therefore, it is crucial to carefully analyze elements that contribute to the inflammatory process when approaching patients with cerebral aneurysms. Furthermore, the presence of wall enhancement on MRI scans can provide additional preoperative assessment, enabling risk stratification based on both inflammatory features and imaging characteristics in intracranial aneurysms. This holistic approach combining clinical, histological, and radiological factors can help guide treatment decisions and improve patient outcomes.

## Data Availability

The original contributions presented in the study are included in the article/Supplementary Material, further inquiries can be directed to the corresponding author.

## References

[1] Vlak MH Algra A Brandenburg R Rinkel GJ. Prevalence of unruptured intracranial aneurysms, with emphasis on sex, age, comorbidity, country, and time period: a systematic review and meta-analysis. Lancet Neurol. (2011) 10:626–36. 10.1016/S1474-4422(11)70109-0 21641282

[2] Brisman JL Song JK Newell DW. Cerebral aneurysms. N Engl J Med. (2006) 355:928–39. 10.1056/NEJMra052760 16943405

[3] Karamanakos PN von Und Zu Fraunberg M Bendel S Huttunen T Kurki M Hernesniemi J Risk factors for three phases of 12-month mortality in 1,657 patients from a defined population after acute aneurysmal subarachnoid hemorrhage. World Neurosurg. (2012) 78:631–9. 10.1016/j.wneu.2011.08.033 22120293

[4] Wermer MJH van der Schaaf IC Algra A Rinkel GJE. Risk of rupture of unruptured intracranial aneurysms in relation to patient and aneurysm characteristics: an updated meta-analysis. Stroke. (2007) 38:1404–10. 10.1161/01.STR.0000260955.51401.cd 17332442

[5] Bederson JB Awad IA Wiebers DO Piepgras D Haley ECJ Brott T Recommendations for the management of patients with unruptured intracranial aneurysms: a statement for healthcare professionals from the stroke council of the American Heart Association. Stroke. (2000) 31:2742–50. 10.1161/01.str.31.11.2742 11062304

[6] Wiebers DO Whisnant JP Huston J 3rd Meissner I Brown RDJ Piepgras DG Unruptured intracranial aneurysms: natural history, clinical outcome, and risks of surgical and endovascular treatment. Lancet. (2003) 362:103–10. 10.1016/s0140-6736(03)13860-3 12867109

[7] Wang G-X Li W Lei S Ge X-D Yin J-B Zhang D. Relationships between aneurysmal wall enhancement and conventional risk factors in patients with intracranial aneurysm: a high-resolution MRI study. J Neuroradiol. (2019) 46:25–8. 10.1016/j.neurad.2018.09.007 30389508

[8] Texakalidis P Sweid A Mouchtouris N Peterson EC Sioka C Rangel-Castilla L Aneurysm formation, growth, and rupture: the biology and physics of cerebral aneurysms. World Neurosurg. (2019) 130:277–84. 10.1016/j.wneu.2019.07.093 31323409

[9] Starke RM Chalouhi N Ali MS Jabbour PM Tjoumakaris SI Gonzalez LF The role of oxidative stress in cerebral aneurysm formation and rupture. Curr Neurovasc Res. (2013) 10:247–55. 10.2174/15672026113109990003 23713738 PMC3845363

[10] Chistiakov DA Killingsworth MC Myasoedova VA Orekhov AN Bobryshev YV. CD68/macrosialin: not just a histochemical marker. Lab Invest. (2017) 97(1):4–13. 10.1038/labinvest.2016.116 27869795

[11] Gottfried E Kunz-Schughart LA Weber A Rehli M Peuker A Müller A Expression of CD68 in non-myeloid cell types. Scand J Immunol. (2008) 67(5):453–63. 10.1111/j.1365-3083.2008.02091.x 18405323

[12] Suzuki H Mikami T Tamada T Ukai R Akiyama Y Yamamura A Inflammation promotes progression of thrombi in intracranial thrombotic aneurysms. Neurosurg Rev. (2020) 43(6):1565–73. 10.1007/s10143-019-01184-3 31686254

[13] Tulamo R Frösen J Hernesniemi J Niemelä M. Inflammatory changes in the aneurysm wall: a review. J Neurointerv Surg. (2018) 10:i58–67. 10.1136/jnis.2009.002055.rep 30037960

[14] Huang MC Baaj AA Downes K Youssef AS Sauvageau E van Loveren HR Paradoxical trends in the management of unruptured cerebral aneurysms in the United States: analysis of nationwide database over a 10-year period. Stroke. (2011) 42:1730–5. 10.1161/STROKEAHA.110.603803 21493902

[15] Gondar R Gautschi OP Cuony J Perren F Jägersberg M Corniola M-V Unruptured intracranial aneurysm follow-up and treatment after morphological change is safe: observational study and systematic review. J Neurol Neurosurg Psychiatry. (2016) 87:1277–82. 10.1136/jnnp-2016-313584 27694497

[16] Virchow R. Ueber die akute entzündung der arterien. Arch Für Pathol Anat Und Physiol Und Für Klin Med. (1847) 1:272–378. 10.1007/BF01975873

[17] Maas U. Die Syphilis als häufigste Ursache der Aneurysmen an der Gehirnbasis. Beitr path Anat. (1936/37) 98:306.

[18] Che J. Molecular mechanisms of the intracranial aneurysms and their association with the long noncoding ribonucleic acid ANRIL—a review of literature. Neurol India. (2017) 65:718–28. 10.4103/neuroindia.NI_1074_15 28681739

[19] Meng H Tutino VM Xiang J Siddiqui A. High WSS or low WSS? Complex interactions of hemodynamics with intracranial aneurysm initiation, growth, and rupture: toward a unifying hypothesis. AJNR Am J Neuroradiol. (2014) 35:1254–62. 10.3174/ajnr.A3558 23598838 PMC7966576

[20] Frösen J Piippo A Paetau A Kangasniemi M Niemelä M Hernesniemi J Remodeling of saccular cerebral artery aneurysm wall is associated with rupture: histological analysis of 24 unruptured and 42 ruptured cases. Stroke. (2004) 35:2287–93. 10.1161/01.STR.0000140636.30204.da 15322297

[21] Kataoka K Taneda M Asai T Kinoshita A Ito M Kuroda R. Structural fragility and inflammatory response of ruptured cerebral aneurysms. A comparative study between ruptured and unruptured cerebral aneurysms. Stroke. (1999) 30:1396–401. 10.1161/01.str.30.7.1396 10390313

[22] Piccirilli M Prizio E Cannizzaro D Tropeano MP Guidetti G Santoro A. The only case of mycotic aneurysm of the PICA: clinical-radiological remarks and review of literature. J Clin Neurosci. (2017) 38:62–6. 10.1016/j.jocn.2016.12.034 28118952

[23] Frösen J Tulamo R Paetau A Laaksamo E Korja M Laakso A Saccular intracranial aneurysm: pathology and mechanisms. Acta Neuropathol. (2012) 123:773–86. 10.1007/s00401-011-0939-3 22249619

[24] Crompton MR. Mechanism of growth and rupture in cerebral berry aneurysms. Br Med J. (1966) 1:1138–42. 10.1136/bmj.1.5496.1138 5932074 PMC1844070

[25] Kaneda K Fujita M Yamashita S Kaneko T Kawamura Y Izumi T Prognostic value of biochemical markers of brain damage and oxidative stress in post-surgical aneurysmal subarachnoid hemorrhage patients. Brain Res Bull. (2010) 81:173–7. 10.1016/j.brainresbull.2009.10.020 19887101

[26] Morel S Diagbouga MR Dupuy N Sutter E Braunersreuther V Pelli G Correlating clinical risk factors and histological features in ruptured and unruptured human intracranial aneurysms: the Swiss AneuX study. J Neuropathol Exp Neurol. (2018) 77:555–66. 10.1093/jnen/nly031 29688417 PMC6005054

[27] Cordina SM Afarian S Gerthoffer WT Martino A Wilson R Naritoku DK. Novel in vivo assessment of unruptured intracranial aneurysm inflammatory factors. Front Neurol. (2020) 11:439. 10.3389/fneur.2020.00439 32582003 PMC7283897

[28] Coen M Burkhardt K Bijlenga P Gabbiani G Schaller K Kövari E Smooth muscle cells of human intracranial aneurysms assume phenotypic features similar to those of the atherosclerotic plaque. Cardiovasc Pathol. (2013) 22(5):339–44. 10.1016/j.carpath.2013.01.083 23466011

[29] Bijlenga P Gondar R Schilling S Morel S Hirsch S Cuony J PHASES score for the management of intracranial aneurysm: a cross-sectional population-based retrospective study. Stroke. (2017) 48(8):2105–12. 10.1161/STROKEAHA.117.017391 28667020

[30] Inci S Akbay A Orunoglu M. Aneurysm clip compression technique in the surgery of aneurysms with hard/calcified neck. World Neurosurg. (2015) 84:688–96. 10.1016/j.wneu.2015.04.039 25931312

[31] Wan J Zhang L Lu G Gu W Huang L Ge L Midterm outcomes of intracranial aneurysms with bleb formation with densely coiling of the aneurismal neck or entire aneurysm. Medicine (Baltimore). (2017) 96:e7046. 10.1097/MD.0000000000007046 28816934 PMC5571671

[32] Song J Liu S Wang T Zhang M Bao G Liang Q The imaging features and endovascular embolization therapy of false aneurysm after the rupture of intracranial aneurysm. Chin J Neurosurg. (2006) 22:741–44.

[33] Juvela S. Growth and rupture of unruptured intracranial aneurysms. J Neurosurg. (2018) 131:843–51. 10.3171/2018.4.JNS18687 30215563

[34] Brown RDJ Broderick JP. Unruptured intracranial aneurysms: epidemiology, natural history, management options, and familial screening. Lancet Neurol. (2014) 13:393–404. 10.1016/S1474-4422(14)70015-8 24646873

[35] Peschillo S Cannizzaro D Missori P Colonnese C Santodirocco A Santoro A Reconstructive endovascular treatment of a ruptured blood blister-like aneurysm of anterior communicating artery. J Neurosurg Sci. (2017) 61(4):438–41. 10.23736/S0390-5616.16.02937-4 24914487

[36] Raymond J Nguyen T Chagnon M Gevry G. Unruptured intracranial aneurysms. Opinions of experts in endovascular treatment are coherent, weighted in favour of treatment, and incompatible with ISUIA. Interv Neuroradiol. (2007) 13:225–37. 10.1177/159101990701300302 20566114 PMC3345486

[37] Morita A Kirino T Hashi K Aoki N Fukuhara S Hashimoto N The natural course of unruptured cerebral aneurysms in a Japanese cohort. N Engl J Med. (2012) 366:2474–82. 10.1056/NEJMoa1113260 22738097

[38] Greving JP Wermer MJH Brown RDJ Morita A Juvela S Yonekura M Development of the PHASES score for prediction of risk of rupture of intracranial aneurysms: a pooled analysis of six prospective cohort studies. Lancet Neurol. (2014) 13:59–66. 10.1016/S1474-4422(13)70263-1 24290159

[39] Etminan N Brown RDJ Beseoglu K Juvela S Raymond J Morita A The unruptured intracranial aneurysm treatment score: a multidisciplinary consensus. Neurology. (2015) 85:881–9. 10.1212/WNL.0000000000001891 26276380 PMC4560059

[40] Can A Castro VM Ozdemir YH Dagen S Dligach D Finan S Alcohol consumption and aneurysmal subarachnoid hemorrhage. Transl Stroke Res. (2018) 9:13–9. 10.1007/s12975-017-0557-z 28752411

[41] Larsen N von der Brelie C Trick D Riedel CH Lindner T Madjidyar J Vessel wall enhancement in unruptured intracranial aneurysms: an indicator for higher risk of rupture? High-resolution MR imaging and correlated histologic findings. AJNR Am J Neuroradiol. (2018) 39:1617–21. 10.3174/ajnr.A5731 30026386 PMC7655285

[42] Shimonaga K Matsushige T Ishii D Sakamoto S Hosogai M Kawasumi T Clinicopathological insights from vessel wall imaging of unruptured intracranial aneurysms. Stroke. (2018) 49:2516–9. 10.1161/STROKEAHA.118.021819 30355091

[43] Hu P Yang Q Wang D-D Guan S-C Zhang H-Q. Wall enhancement on high-resolution magnetic resonance imaging may predict an unsteady state of an intracranial saccular aneurysm. Neuroradiology. (2016) 58:979–85. 10.1007/s00234-016-1729-3 27438805

[44] Lv N Karmonik C Chen S Wang X Fang Y Huang Q Relationship between aneurysm wall enhancement in vessel wall magnetic resonance imaging and rupture risk of unruptured intracranial aneurysms. Neurosurgery. (2019) 84:E385–91. 10.1093/neuros/nyy310 30011026

[45] Liu P Qi H Liu A Lv X Jiang Y Zhao X Relationship between aneurysm wall enhancement and conventional risk factors in patients with unruptured intracranial aneurysms: a black-blood MRI study. Interv Neuroradiol. (2016) 22:501–5. 10.1177/1591019916653252 27341856 PMC5072207

[46] Wang X Zhu C Leng Y Degnan AJ Lu J. Intracranial aneurysm wall enhancement associated with aneurysm rupture: a systematic review and meta-analysis. Acad Radiol. (2019) 26:664–73. 10.1016/j.acra.2018.05.005 29908979

[47] Texakalidis P Hilditch CA Lehman V Lanzino G Pereira VM Brinjikji W. Vessel wall imaging of intracranial aneurysms: systematic review and meta-analysis. World Neurosurg. (2018) 117:453–8.e1. 10.1016/j.wneu.2018.06.008 29902602

